# The Impact of Mutation L138F/L210F on the Orai Channel: A Molecular Dynamics Simulation Study

**DOI:** 10.3389/fmolb.2021.755247

**Published:** 2021-11-02

**Authors:** Xiaoqian Zhang, Hua Yu, Xiangdong Liu, Chen Song

**Affiliations:** ^1^ Center for Quantitative Biology, Academy for Advanced Interdisciplinary Studies, Peking University, Beijing, China; ^2^ School of Physics, Shandong University, Jinan, China; ^3^ College of Plant Protection, Shandong Agricultural University, Taian, China; ^4^ Peking-Tsinghua Center for Life Sciences, Academy for Advanced Interdisciplinary Studies, Peking University, Beijing, China

**Keywords:** orai, mutation, gating, ion channel, molecular dynamics

## Abstract

The calcium release-activated calcium channel, composed of the Orai channel and the STIM protein, plays a crucial role in maintaining the Ca^2+^ concentration in cells. Previous studies showed that the L138F mutation in the human Orai1 creates a constitutively open channel independent of STIM, causing severe myopathy, but how the L138F mutation activates Orai1 is still unclear. Here, based on the crystal structure of *Drosophila melanogaster* Orai (dOrai), molecular dynamics simulations for the wild-type (WT) and the L210F (corresponding to L138F in the human Orai1) mutant were conducted to investigate their structural and dynamical properties. The results showed that the L210F dOrai mutant tends to have a more hydrated hydrophobic region (V174 to F171), as well as more dilated basic region (K163 to R155) and selectivity filter (E178). Sodium ions were located deeper in the mutant than in the wild-type. Further analysis revealed two local but essential conformational changes that may be the key to the activation. A rotation of F210, a previously unobserved feature, was found to result in the opening of the K163 gate through hydrophobic interactions. At the same time, a counter-clockwise rotation of F171 occurred more frequently in the mutant, resulting in a wider hydrophobic gate with more hydration. Ultimately, the opening of the two gates may facilitate the opening of the Orai channel independent of STIM.

## Introduction

Calcium ions, as an essential second messenger in cells, regulate a wide range of physiological processes. Store-operated calcium entry (SOCE) was identified to explain how depletion of endoplasmic reticulum (ER) Ca^2+^ stores evokes Ca^2+^ influx across the plasma membrane. (Putney, 1986). Up to now, the relatively well-studied SOCE channel is the “calcium release-activated calcium” (CRAC) channel, which is involved in numerous cell activities such as gene transcription, muscle contraction, secretion, cell proliferation, differentiation and apoptosis etc. (Engh et al., 2012; Feske et al., 2012; Soboloff et al., 2012; Prakriya and Lewis, 2015) Both loss-of-function and gain-of-function mutations of the CRAC channel lead to devastating immunodeficiencies, bleeding disorders and muscle weakness. (Feske et al., 2005; Feske, 2010; Endo et al., 2015; Garibaldi et al., 2017). In recent decades, our understanding of the operational mechanisms of the CRAC channel including the gating mechanism has been greatly advanced, with the discovery of its molecular components, stromal interaction molecule (STIM) and the pore-forming protein Orai. (Liou et al., 2005; Prakriya et al., 2006). The STIMs are single-pass ER transmembrane proteins, function as the sensor of the Ca^2+^ concentration inside the ER, bind to and activate Orai channels. (Liou et al., 2005; Stathopulos et al., 2006). Two mammalian homologs, STIM1 and STIM2, are included in the STIMs family and the former one is more widely studied. Orai, the calcium channel that opens to permit the influx of the calcium ions, locates on the plasma membrane and contains three closely conserved mammalian homologs, Orai1, Orai2 and Orai3. (Vig et al., 2006; Hoth et al., 2013).

**FIGURE 1:**
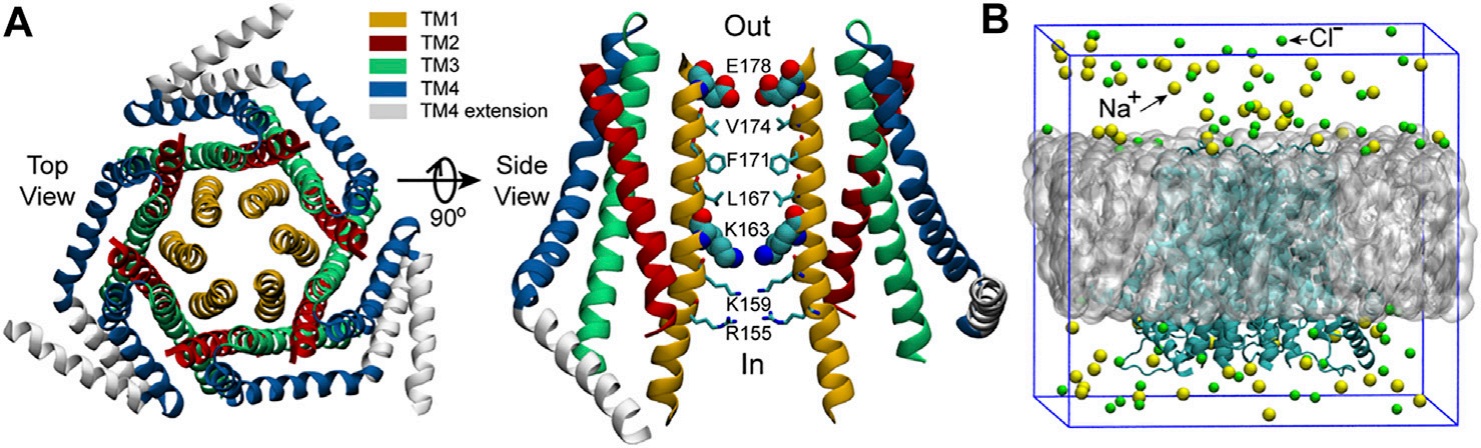
The structure of dOrai and the initial simulation system. **(A)** The crystal structure of *Drosophila melanogaster* Orai obtained from the Protein Data Bank (PDB ID: 4HKR) (Hou et al., 2012). In the right panel, only two oppositing subunits are shown for clarity. The SF of the pore, E178, and the starting residue of the basic region, K163, are shown with the VDW representation, the other pore residues are shown with Licorice representation in VMD (visual molecular dynamics) (Humphrey et al., 1996). **(B)** The initial simulation system. The grey sphere represents the POPC bilayer. Water molecules are not shown here but included in the simulations.

Orai1 has a high calcium selectivity (>1000-fold over Na^+^) and low conductivity (<1 pS). (Hogan et al., 2010; Prakriya and Lewis, 2006). According to the structure of Orai (Figure 1A), (McNally et al., 2009; Zhou et al., 2010; Hou et al., 2012) the transmembrane Orai is composed of six subunits with a central pore formed by six helices denoted as transmembrane one (TM1). TM1 are surrounded by two rings: one is composed of TM2 and TM3, the other is TM4. There is another helix which extends into the cytosol, termed TM4 extension. As TM1 helices are tightly wrapped by TM2 and TM3 helices, they may have limited space to expand to allow the CRAC channel open. (Liou et al., 2005; Roos et al., 2005). The TM1 helices can be divided into four distinct regions (Figure 1A): the selectivity filter (SF) - a ring of glutamates (E178), the hydrophobic region (V174, F171, L167), the basic region (K163, K159, R155) and the cytosolic region. (Hou et al., 2012). The glutamate-ring (E178) functions as a SF and makes the channel have a high calcium ion selectivity, which is the most significant feature of Orai channels. Mutation of the residue E178 to aspartate disrupts Ca^2+^-selectivity. (Yeromin et al., 2006). The well-packed side chains of V174, F171 and L167 form the inner wall of the hydrophobic region, having extensive hydrophobic interactions with one another, and are strictly conserved among Orai channels. (McNally et al., 2012; Gudlur et al., 2014). These hydrophobic residues are located at the center of the protein, which likely form a gate of the pore. The V174A mutation yields an activated channel with altered ion selectivity even if its pore structure shows no obvious changes compared to the wild-type (WT), and a slight difference of the number of water molecules in the hydrophobic region is enough to change the conduction state of the pore, (Dong et al., 2013), indicating the significant role of the hydrophobic region in gating. Another important region locates in the lower part of the channel and lines by three basic residues (K163, K159 and R155), creating an unexpected positively charged environment for the pore that conducts cations. Generally, K163 corresponds to the narrowest point of the pore, resulting in large electrostatic repulsion between this positively charged residue and cations passing by. Therefore, K163 is believed to be the other gate of the pore and jointly regulates the channel state together with the hydrophobic gate. (Zhang et al., 2011).

Molecular dynamics (MD) simulation is a powerful tool to study the gating and permeation mechanisms of ion channels, which can provide detailed dynamic information. Based on the crystal structure of the closed dOrai (Hou et al., 2012), many MD works have been done to understand the gating and permeation processes through simulations of the WT dOrai/hOrai1 (Orai1 of human), or dOrai/hOrai1 mutants that were either constitutively open or loss-of-function. Mutations on the TM1 were the first to get attention as TM1 constitutes the pore. Through simulations of the dOrai V174A mutant, Dong et al. revealed the regulation of pore waters to the ion permeation and the counterion-assisted cation transport mechanism of Orai. (Dong et al., 2013; Dong et al., 2014). Through simulations for the dOrai V174A, F171V and F171Y mutants, Yamashita et al. revealed the counter-clockwise rotation of F171. (Yamashita et al., 2017). The hOrai1 E106D mutant (corresponding to dOrai E178D mutant) was used to study the binding site of the selective inhibitor Synta66. (Waldherr et al., 2020). These simulations were all performed with CHARMM36/27 (MacKerell et al., 1998) force field. Mutations on the TM2, such as hOrai1 H134A, L138F, A137V and R91G (TM1) (corresponding to dOrai H206A, L210F, A209V and R163G mutants) were investigated to show the regulation of TM2 to the channel states through transmembrane helix connectivity, particularly via the hydrogen bonding in the H134A mutant. (Frischauf et al., 2017). Mutations on the extracellular loops (loop1 between TM1 and TM2, and loop3 between TM3 and TM4), such as hOrai1 D110A, R210A, K214A and R210A/K214A, were systematically studied, which revealed the presence of an extracellular Ca^2+^-accumulating region at the pore entrance of hOrai1. (Frischauf et al., 2015). These above two work were performed with the OPLS (Tirado-Rives, 1988) (Optimized Potentials for Liquid Simulations) all-atom force field. In addition, the pore hydration and degrees of the counter-clockwise rotation of F171 were also investigated in several TM2 mutants (dOrai H206S/C/Q/Y mutants) with CHARMM36 force field. (Yeung et al., 2018). Through conventional molecular dynamics with CHARMM36 force field and Brownian dynamics, a mutation on TM3 (dOrai E262Q) was studied, which revealed the key role of the configuration of residues K270 on the selectivity of the pore. (Alavizargar et al., 2018). Besides, a series of double point mutants that involve both a gain-of-function and a loss-of-function single point mutations were studied, which confirmed the dominant role of loss-of-function mutations (hOrai1 K85E/H134A, H134A/E149K, H134A/L174D and H134A/S239W mutants, corresponding to dOrai K157E/H206A, H206A/E221K, H206A/L246D and H206A/S311W mutants). (Tiffner et al., 2021). Multiple point mutants were also investigated. A series of multiple point mutations on basic residues were studied and revealed the promotion of the inner basic residues to the opening of the outer hydrophobic gate (dOrai R155S/K159S/R163S, R155S/K159S/R163S/V174A, R155S/K159S/R163S/W148A, K159S/K163S/V174A, K159S/K163S, K163W/V174A and K163W mutants). (Yamashita et al., 2019). This work was also performed with CHARMM36 force field.

The aforementioned computational studies have provided highly valuable insight for understanding the gating and permeation mechanisms of Orai channels. In the meantime, previous experiments have also shown that the L138F mutation in human Orai1 yields a constitutively permeant channel that allows ion conduction in the absence of STIM1, and the constitutively active L138F mutant channel can cause severe myopathy. (Endo et al., 2015). However, the activation mechanism of the L138F Orai1 mutant is not well studied yet. The structure of human Orai1 has not been resolved so far, while the closed state crystal structure of dOrai, which shares 73% sequence identity with human Orai1 within the transmembrane region, has been resolved by Hou et al. at a resolution of 3.35 Å (Figure 1A). (Hou et al., 2012) The L138F mutation in human Orai1 corresponds to the mutation L210F in dOrai of *Drosophila melanogaster*. In order to better understand how L210F mutation activates the channel, molecular dynamics (MD) simulations for the WT and the L210F mutant channels were carried out. Our results revealed a previously unobserved rotation of the residue F210 in the mutant, and the larger rotation angle of F210 in the mutant might be the origin of the activation of the L210F mutant. It was also observed that the rotation of F171 may also play an important role for the activation, as previously reported (Yamashita et al., 2017). Therefore, our simulation results reveal a plausible activation mechanism of the L210F dOrai mutant and may provide a new perspective for understanding the activation mechanism of CRAC channels.

## Materials and Methods

### Molecular Dynamics Simulations

The crystal structure of *Drosophila melanogaster* Orai obtained from the Protein Data Bank (PDB ID: 4HKR) (Hou et al., 2012) was used as the starting structure. MODELLER (Fiser et al., 2000) was used to build the missing residues of the TM1-TM2 loop (residue number: 181–190) and the TM2-TM3 loop (residue number: 220–235) on the basis of the starting structure. After that, the complete WT dOrai structure was used to construct the initial simulation system of the WT dOrai (Figure 1B) using the bilayer-builder module of CHARMM-GUI (Jo et al., 2010) with the channel axis oriented along the *z*-axis. The above complete WT dOrai structure was also used to construct the initial simulation system of the L210F dOrai mutant (Figure 1B) in a very similar way, except that in the PDB Info section, the mutation option under PDB manipulation was chosen and L210 of all the six chains were set to mutate to F210 in CHARMM-GUI. For each system, the protein was embedded within a 1-palmitoyl-2-oleoyl-sn-glycero-3-Phoss-ph-ocholine (POPC) bilayer with 150 mM NaCl to neutralize the system. The final system size was 110.3 × 110.3 × 105.3 Å^3^ and there were around 116k atoms in the simulation system.

All the molecular dynamics simulations were conducted using GROMACS 5.1.3 (Abraham et al., 2015) with CHARMM36 (MacKerell et al., 1998; Klauda et al., 2010; Best et al., 2012) force field and TIP3P (Jorgensen et al., 1983) water model. A 500-ps NVT equilibration and a 500-ps NPT equilibration were performed after energy minimization. Then, three 500-ns production simulations with different starting velocities were conducted for each system. Position restraints with a force constant of 1,000 kJ/mol/nm^2^ (Soboloff et al., 2012) were applied on the backbone atoms of the protein for the equilibration simulations. The periodic boundary conditions were used and the time step was 2 fs. The velocity-rescaling algorithm (Bussi et al., 2007) with a time constant of 0.5 ps was used to maintain the temperature at 310 K. Protein, membrane, and water and ions were coupled separately. The Parrinello-Rahman algorithm (Parrinello and Rahman, 1981) with a time constant of 5 ps was used to maintain the pressure at 1.0 Bar. The Particle-Mesh-Ewald (PME) (Essmann et al., 1995) method was used to calculate electrostatics and the van der Waals interactions were computed within a cut-off of 1.2 nm. VMD (Humphrey et al., 1996) was used to view trajectories and render figures.

## Results

### Structural Statibilty of the WT Orai and the L210F Mutant

**FIGURE 2:**
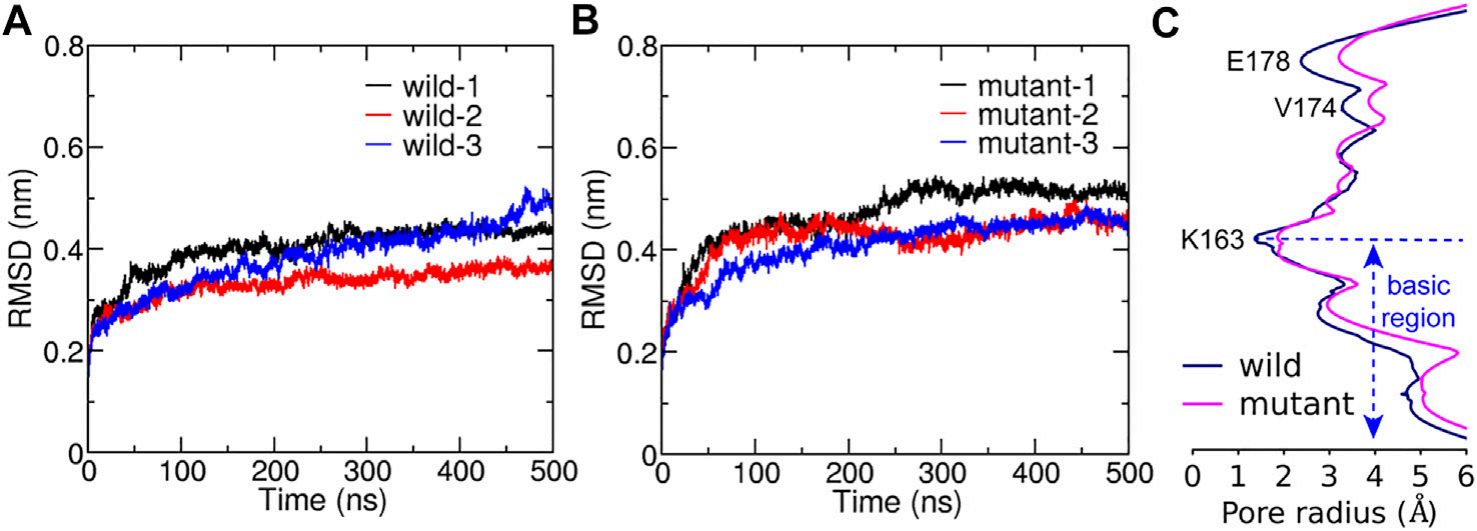
The structural stability during simulations and the average pore radius after reaching equalibrium. C α -RMSD of the WT **(A)** and the L210F mutant **(B)**. Three trajectories are represented in red, blue and black, respectively. **(C)** The average pore radii. The average pore radii for the WT and the L210F mutant were obtained by calculating the pore radius of the average structures obtained from the last 200 ns of all of the three trajectories for each protein. Hole 2.0 (Smart et al., 1996) was used to calculate the radius with all the hydrogen atoms removed in the calculation.

The root mean square deviation (RMSD) of all six trajectories for both the WT and the L210F mutant were monitored to evaluate their structural changes and stability. The results showed that the systems reached equilibrium states at about 300 ns (Figures 2A,B) with the C α -RMSD of the WT and the mutant converging to 0.41 and 0.47 nm respectively. This time scale and the RMSD values were slightly larger than the work of Amcheslavsky et al. (Amcheslavsky et al., 2015) and Frischauf et al. (Frischauf et al., 2017), in which the values were roughly 120 ns and 0.2 nm. As Amcheslavsky et al. (Amcheslavsky et al., 2015) discussed, the smaller RMSD in their work may be caused by different system setups that can stabilize the protein structure, such as the addition of phosphates in the basic region, using of neutralized protonation states, addition of cation ions (Ca^2+^ or Gd^3+^) in the SF and addition of cholesterol into the membrane. Our results were more comparable to the work of Dong et al. (Dong et al., 2013) owing to similar simulation setups, indicating that the stability of Orai can be influenced by the surrounding environment. In addition, the RMSD of the mutant experienced a larger increase during the initial stage of the simulations, reflecting the influence of the residue mutation on the structure (Figure 2B).

The pore radius was calculated to evaluate the effect of the L210F mutation on the channel state (Figure 2C and Supplementary Figure S1). The simulation results revealed three significant radius changes of the mutant (Figure 2C). The first one occurred in the SF, the glutamate ring - E178, which binds and transports Ca^2+^ selectively. The pore radius at E178 of the L210F mutant expanded by approximately 1 Å compared with the WT channel. An increase in the radius of the SF may increase the chance of ion binding and thus improve the probability of ions entering into the pore for the mutant. The second change was at the starting residue of the hydrophobic region, V174. The dilation of V174 in the mutant may allow more water molecules to stay at this entrance of the hydrophobic region, which lays a good foundation for water to further occupy the following hydrophobic region. The third change occurred in the basic region, where nearly the whole segment of the mutant was wider than the WT. The increase of the basic region radius can not only reduce the steric hindrance, but also reduce the electrostatic exclusion between the basic residues and cation ions passing through this cationic channel. Moreover, the constriction site of the channel, the K163 gate located at the beginning of the basic region, expanded significantly in the mutant channel (Figure 2C), which might be a key step for the activation of the L210F mutant.

### The Rotation of the Residue L/F210

**FIGURE 3:**
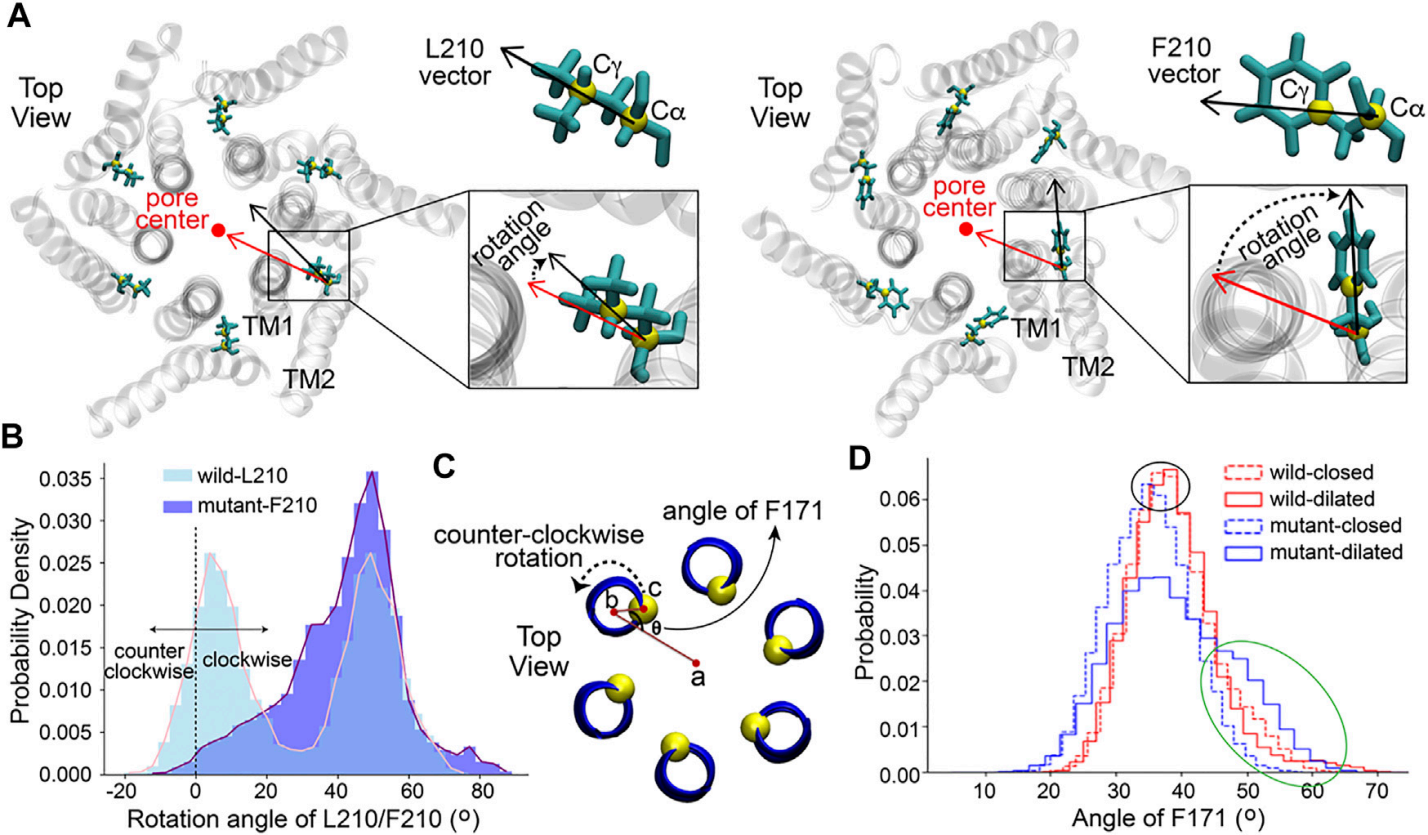
The rotation of residues L/F210 and F171. **(A)** The definition of the rotation angle of L/F210. The rotation angle was defined by the black and red vectors. The red vector was determined by the projections of C α of residue 210 and the pore center onto the XY plane, and the pore center was the geometry center of C α atoms of all the six TM1s. The black vectors of L/F210 were determined by the projection of C α and C γ onto the XY plane. Only the TM1 and TM2 in each subunit are shown for clarity. One frame of the simulation is shown with gray transparent NewCartoon as an example. L210 and F210 are shown with Licorice. **(B)** The distribution of the rotation angle of L/F210. The last 200 ns of each trajectory was used for this analysis. VMD was used for the calculations. **(C)** The definition of the angle of F171 (top view). Point a was obtained by projecting the center of the channel on the XY plane. Similarly, point b was determined by the geometry center of two helices centered on F171 (residues 169–173), and point c was Cα of F171. The axis of rotation passed through point b and was parallel to the channel axis. **(D)** The distribution of the angle of F171. The four data sets, wild-closed, wild-dilated, mutant-closed and mutant-dilated, were classified with a pore radius of 2 Å at the K163 gate. 500 ns of each trajectory was used for the analysis. VMD was used for the calculations.

A rotation angle defined by two vectors, which were determined by two carbon atoms of residue 210 and the pore axis, was calculated to evaluate the rotation of the residue around its rotation axis that is parallel to the *z*-axis and passes through Cα of residue 210 (detailed definition in Figure 3A). The results revealed a previously unobserved rotation of the residue L/F210. L210 in the WT channel had two populated distributions of the rotation angle (Figure 3B). The peaks of the two distributions were at about 5 and 50°, respectively. In constrast to L210, only one major distribution with a higher peak located at about 50° was observed for F210 in the mutant channel (Figure 3B). Therefore, it appeared that L210 could have two major conformations, in which the side chain of L210 either points to the pore axis or rotates clockwise for about 50°. The two conformations are equally stable as they show nearly identical distribution and free energies (Supplementary Figure S2). In the mutant, F210 showed only one major conformation, in which it prefered to rotate clockwise for about 50° owing to the lowest free energy (Supplementary Figure S2). F210 also showed larger maximum rotation compared to L210, reaching 80–90° where F210 would be pointing to a nearly tangential direction of the pore. These conformations with large rotation angles kept F210 farther away from the pore-lining helix (TM1), which will probably generate a pulling effect on the TM1 through hydrophobic interactions with A166 on the TM1 of the same subunit (Supplementary Figure S3) and leave more room for the TM1 to expand outward. This may be the reason that caused the expansion of the basic region located in the TM1 adjacent to F210 in the mutant channel (Supplementary Figure S3).

### The Counter-Clockwise Rotation of the Residue F171

It was reported that the opening of the Orai channel is accompanied by the counter-clockwise rotation of the residue F171 (Yamashita et al., 2017), which is located on the TM1 in the middle of the hydrophobic gate and is some distance away from the mutation point L/F210 (Figure 1A and Supplementary Figure S3). The presence of the hydrophobic gate increases the energy barrier of ion permeation, while the rotation of the residue F171 may reduce this barrier, contributing to the activation of the channel (Yamashita et al., 2017). Here, the orientation angle of F171 (definition (Yamashita et al., 2017) in Figure 3C) was calculated to measure the dynamics of this residue in our simulations. Firstly, in order to investigate the angle of F171 in a more detailed pore radius range, the structures of each channel obtained from MD simulations were classified into two classes according to the pore radius at the K163 gate, which is the constriction site of the channels. Structures with a radius at K163 of less than 2 Å were classified as the closed state while structures with a K163 radius of more than 2 Å were classified as the dilated state. As a result, four data sets: wild-closed (10,123 frames), wild-dilated (4,877 frames), mutant-closed (5,175 frames) and mutant-dilated (9,825 frames) were obtained. The results of the angle showed a very similar distribution among the wild-closed, wild-dilated and mutant-closed data sets with the most frequent angles of 35–40° (Figure 3D, the black oval). These distributions were supposed to be caused by the normal fluctuations of F171. However, the angle distribution of the mutant-dilated data set was different, with a probability increase of angles above 45° (Figure 3D, the green oval, about 45–65°) and a probability decrease of angles around 35–40°. A larger rotation angle of F171 will keep this residue pointing away from the pore axis, which will further allow more hydration at this site. This appears to be caused by the dilation of V174, on the basis of the structural change at F210 in the mutant (Figure 2C), and will lead to a more open hydrophobic gate. Therefore, the angles above 45° were believed to make contributions to the opening of the hydrophobic gate of the mutant. Then, the effective counter-clockwise rotation of F171 that might open the hydrophobic gate was about 10–30° (45\65 minus 35) after eliminating its normal fluctuations.

### Water in the Pore

**FIGURE 4:**
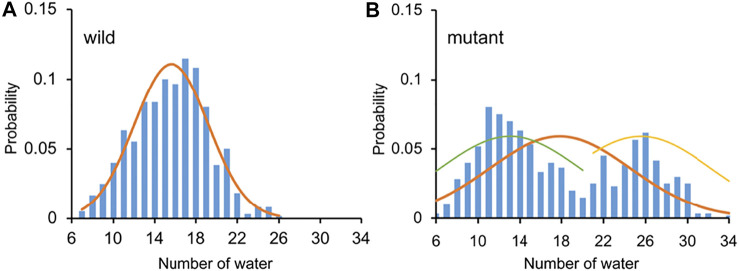
The number of water molecules and the corresponding probability in the pore of the WT **(A)** and the L210F mutant **(B)** channels. From residue F171 to E178 along the central axis of the pore, the number of water molecules (oxygen atoms were measured) within a cylinder of 5-Å radius was calculated using VMD for the last 200 ns of all three trajectories for each protein system.

The Orai channel has two gates, the residue K163 gate in the basic region and the hydrophobic gate (residues F171-V174). Previous studies have shown that even if the radius of the Orai channel does not significantly change, a limited increase of hydration in the pore is enough to regulate the conduction state (Dong et al., 2013), suggesting the importance of the hydrophobic gate. Hence, the number of water molecules in the pore, as an important indicator of the conductivity of the channel, was calculated to measure the hydration difference between the WT and the L210F mutant channels. The water distributions in the region from residues F171 to E178 were measured. This region includes the hydrophobic region and the SF, which was reported to be the area of the most significant water distribution difference. (Dong et al., 2013). The results showed that the distribution of the number of water molecules was relatively concentrated with the average number of 16 in the pore of the WT (Figure 4A). However, the distribution showed two peaks in the L210F mutant (Figure 4B). A low-hydration distribution and a high-hydration distribution were observed, and the average number for the whole distribution was about 18 (Figure 4B), larger than the average number in the WT. Besides, the mutant can have a hydration number as high as ∼30, a value the WT channel never reached. Accordingly, the highly hydrated structures of the L210F mutant were supposed to reduce the energy barrier of ions passing through the hydrophobic gate and thus facilitate ion permeation.

### Na^+^ in the Pore

**FIGURE 5:**
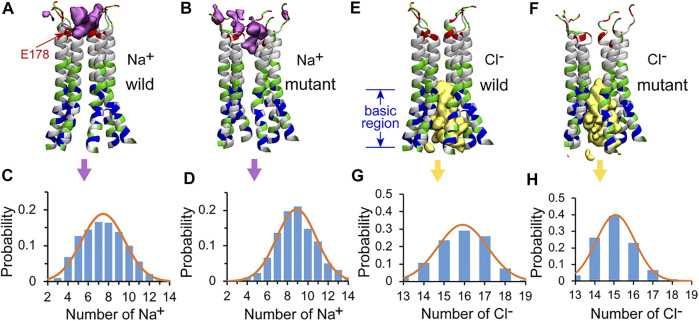
The distributions of Na^+^ and Cl^−^ in the pore of the channel. The isosurface of Na^+^ ion density in the WT **(A)** and the L210F mutant **(B)** channels. The number of Na^+^ ions and corresponding probability in the pore of the WT **(C)** and the L210F mutant **(D)** channels. The isosurface of Cl^−^ ion density in the WT **(E)** and the L210F mutant **(F)** channels. The number of Cl^−^ ions and corresponding probability in the pore of the WT **(G)** and the L210F mutant **(H)** channels. The isosurface of Na^+^ and Cl^−^ ion densities are shown in pink and yellow with isosurface values of 0.02 and 0.06, respectively. Only residues from W148 to D184 are shown with NewCartoon and colored by residue types for clarity. Blue, basic residues; red, acidic residues; green, polar residues; white, nonpolar residues. From residue W148 to D184 along the central axis of the pore, the number of Na^+^ and Cl^−^ ions within a cylinder of 10-Å radius were calculated using VMD for the last 200 ns of all three trajectories for each protein system.

Although the Orai channels are highly selective for Ca^2+^, Na^+^ was often used in the study of the conductivity of CRAC channels as Na^+^ can permeate at a much higher rate in the absence of Ca^2+^, (Prakriya and Lewis, 2006; Hogan et al., 2010), which makes it easier for better sampling in MD simulations. The position and the number of Na^+^ ions within the pore were calculated to observe the behavior of Na^+^ ions in the WT and the L210F mutant channels. The results showed that Na^+^ ions were mainly distributed in the upper part of the pore (Figures 5A,B). Three binding sites, residues E178, D182 and D184, were observed (Supplementary Figure S4), among which E178 was the most dominant one. Na^+^ ions permeated deeper in the mutant than in the WT (below E178 in the mutant and a little above E178 in the WT, Figures 5A,B), and the most frequent numbers of Na^+^ ions in this region were 7 and 9 for the WT and the mutant, respectively (Figures 5C,D). Therefore, it seems that the L210F mutation moderately modified the distribution of Na^+^ ions along the pore, causing more Na^+^ accumulation at the entrance of the pore and increasing the probability of ions passing through, which is consistent with the fact that the L210F mutant is constitutively open to cations. However, no spontaneous Na^+^ permeation was observed in our MD process, probably due to the extremely low conductivity of the L138F Orai1 (equals to the L210F dOrai here) mutant (Frischauf et al., 2017) and the lacking of a fully open structure.

### Cl^−^ in the Pore

As previous studies showed anion-assisted cation permeation in the V174A Orai channel (also a constitutively open channel), (Dong et al., 2014), the position and number of Cl^−^ ions within the pore were calculated to analyze the behavior of Cl^−^ ions in the WT and the L210F mutant channels in our simulations as well. The results showed that Cl^−^ ions were mainly distributed in the intracellular region, specifically referring to the basic region including residue K163 and the residues below it (Figures 5E,F). The most frequent numbers of Cl^−^ ions were 16 and 15 in the WT and the mutant channels, respectively (Figures 5G,H), showing no appreciable difference. Previous molecular dynamics simulations reported that Cl^−^ ions in the basic region of the open V174A mutant can flow out to the extracellular side of the pore under electric field conditions, coordinating with Na^+^ ions in the pore to form an energetically more favorable cluster to help the influx of Na^+^ ions. (Dong et al., 2014). It seems that the mutation L210F does not alter the Cl^−^ occupation around the basic region or its role in assisting cation permeation.

## Discussion

In this paper, based on the crystal structure of *Drosophila melanogaster* Orai, we investigated the detailed channel structures and the water and ion distributions for both the WT and the L210F mutant channels by using molecular dynamics simulations. The results revealed two small but essential conformational changes resulting from the L210F mutation. Firstly, an previously unobserved rotation of residue F210 initiated the channel opening in the L210F mutant. F210 in the mutant had larger outward rotation than L210 in the WT, resulting in the dilation of the basic region and the K163 gate. At the same time, a 20-degree (on average) counter-clockwise rotation of F171 in the hydrophobic gate occurred more frequently and allowed more hydration at the hydrophobic region, which can potentially lead to the opening of the hydrophobic gate. Collectively, the rotation of F210 and F171, leading to the opening of the two gates of the channel, may create a constitutively open L210F mutant. Therefore, our results may shed further light on the disease of myopathy caused by the L138F mutation in human Orai1, by providing insight into the detailed structure and activation mechanism of the L210F mutant.

This is the first time that the rotation of the residue 210 is characterized to be the key origin of the activation for the L210F mutant channel. In contrast to the WT L210 that has two equally distributed rotation angles, the mutant F210 showed a predominantly larger clockwise rotation, which leaves more room for the TM1 helices to expand outward, dilating the basic region and the K163 gate. The expansion of the basic region involved hydrophobic interactions between TM2 and TM1 in the same subunit, suggesting the important role of the transmembrane helix (TH) interaction network on the channel gating. Apparently, the regulation of the TH network may work in more than one way. In Frischauf’s work for another constitutively open H134A Orai1 mutant (equals to the H206A dOrai mutant), the regulation of the TH connectivity on the channel gating is shown in the disruption of hydrogen bonds between H134 on the TM2 and two residues on the TM1 (S93 and S97). (Frischauf et al., 2017). Notably, the L138F Orai1 mutant (equals to the L210F dOrai here) is also studied through molecular dynamics simulations in the same work, which showed that the enhanced hydrophobic contacts between TM2 and TM1 through L138F mutation trigger more flexibility of the pore, and then one water chain enters into the hydrophobic region and opens the pore. (Frischauf et al., 2017). These results are generally consistent with ours, both of which emphasize the importance of the hydrophobic interaction between TM2 and TM1 and water chain or hydration in the hydrophobic region. However, our results revealed a previously unnoticed rotation of residue 210, which may be the origin of the activation. Apart from the rotation of the residue 210, the rotation of F171 observed in our simulation, which was also reported to be required in other constitutively open channels V174A and F171Y in Yamashita’s work (Yamashita et al., 2017), may also be an important factor for gating.

Notably, no significant rotational movement of F171 or TM1 was observed from the open conformation of the H206A dOrai (equals to the H134A Orai1) resolved at 3.3 Å resolution by cryo-EM recently (Hou et al., 2020), indicating that multiple activation mechanisms may be utilized by different mutants. The regulation of the TH network is also shown in the dilation of the filter. TM1, TM2 and TM3 in two adjacent subunits may participate in this process (Supplementary Figure S5). The residue K270 on the TM3 was reported to regulate the filter selectivity through conformation dynamics and the filter dilation was also observed in the previous study. (Alavizargar et al., 2018). In our study, the radius of the K270 ring was dilated in the L210F mutant (the average radius of K270 ring in the WT and the mutant: 12.30 Å and 13.33 Å), which was probably caused by the expansion of the filter through electrostatic interactions (Supplementary Figure S5). However, how the mutation of residue 210 allosterically affects K270 is still not clear. Further simulations and analysis are undergoing to focus on the selectivity difference between the WT and the L210F mutant using a new calcium model, (Zhang et al., 2020), which will hopefully provide a better understanding on this aspect.

The dilation of the entire pore obtained in our simulations for the L210F mutant is consistent with the recently obtained open-state cryo-EM structure of the H206A dOrai mutant (Hou et al., 2020). Both H206A and L210F mutations occur on the TM2 and construct constitutively open channels with some selectivity for Ca^2+^ remained, so some structural similarity of the pore may exist among the H206A mutant, the L210F mutant and the WT Orai. (Frischauf et al., 2017). The dilation of the filter in the open channels didn’t get much attention previously. In some previous simulations (Frischauf et al., 2017), the filter of the constitutively open channel is nearly the same with the closed Orai, since Ca^2+^ were trapped in the filter in both simulations owing to strong interactions between Ca^2+^ and protein. Here, we didn’t include Ca^2+^ in our simulations, following the protocol of other previous simulations (Dong et al., 2013), and therefore the filter was more flexible to reach a more dilated state in the mutant, in agreement with the filter dilation as observed in the open-state H206A structure (Hou et al., 2020). In addition, it was reported that the WT Orai1 experiences some filter conformational changes when it is activated by STIM1 (Gudlur et al., 2014), suggesting that the filter conformation may be different for the open channel and the closed channel. Still, it should be noted that both the L210F Orai mutant and the H206A cyro-EM structure may not fully mimic the STIM-dependent Orai gating since their conductivity and selectivity are not the same after all.

## Data Availability

The original contributions presented in the study are included in the article/Supplementary Material, further inquiries can be directed to the corresponding authors.
