# Supplementary material for: The Impact of Mutation L138F/L210F on the Orai Channel: A Molecular Dynamics Simulation Study
Source: Front Mol Biosci. 2021 Nov 2;8:755247. doi: 10.3389/fmolb.2021.755247 (PMC8592927; doi:10.3389/fmolb.2021.755247)
Supplement: Supplementary file 1 [file DataSheet1.pdf]

Supplementary Information for:

The impact of mutation L138F/L210F on the Orai channel: a molecular dynamics simulation study

Xiaoqian Zhang[1,2†], Hua Yu[1,3†*], Xiangdong Liu[2], and Chen Song[1,4*]

[1]Center for Quantitative Biology, Academy for Advanced Interdisciplinary Studies, Peking University, Beijing 100871, China

[2]School of Physics, Shandong University, Jinan 250100, Shandong Province, China

[3]College of Plant Protection, Shandong Agricultural University, Taian 271018, Shandong Province, China

[4]Peking-Tsinghua Center for Life Sciences, Academy for Advanced Interdisciplinary Studies, Peking University, Beijing 100871, China

[†]These authors contributed equally to this work.

[*]For correspondence: yuh@sdau.edu.cn (HY); c.song@pku.edu.cn (CS)

Supplementary Figures

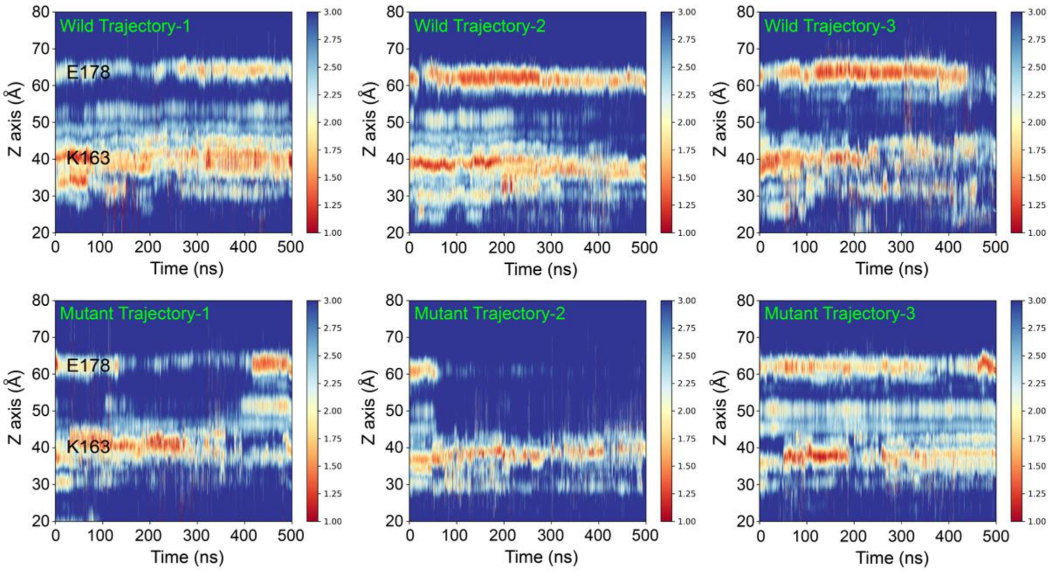

Figure S1. Pore radius evolution of the six trajectories. The pore radius was calculated using HOLE 2.0.

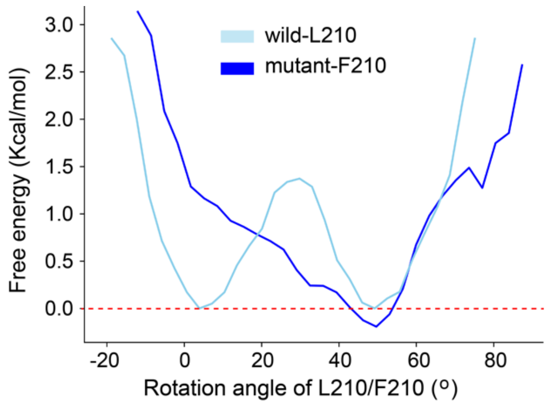

Figure S2. Conformational free energy of L/F210. This was calculated according to the probability density of L/F210 (Fig. 3B).

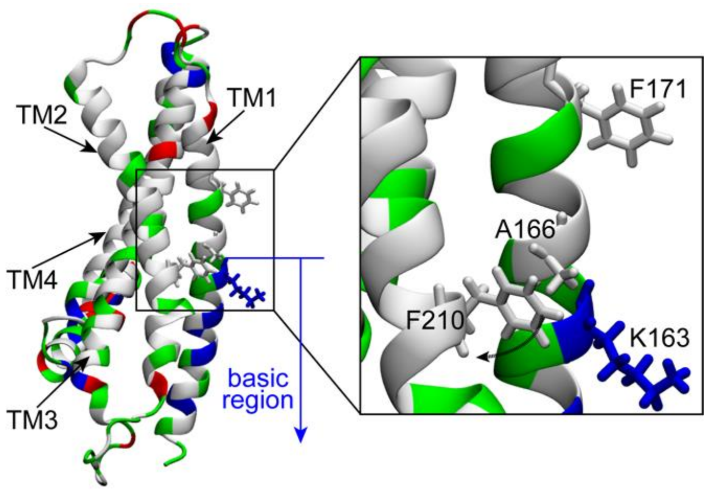

Figure S3. The location of F210, A166, K163 and F171. The outward rotation of F210 might be the reason of the dilation of the basic region. Only one subunit is shown for clarity.

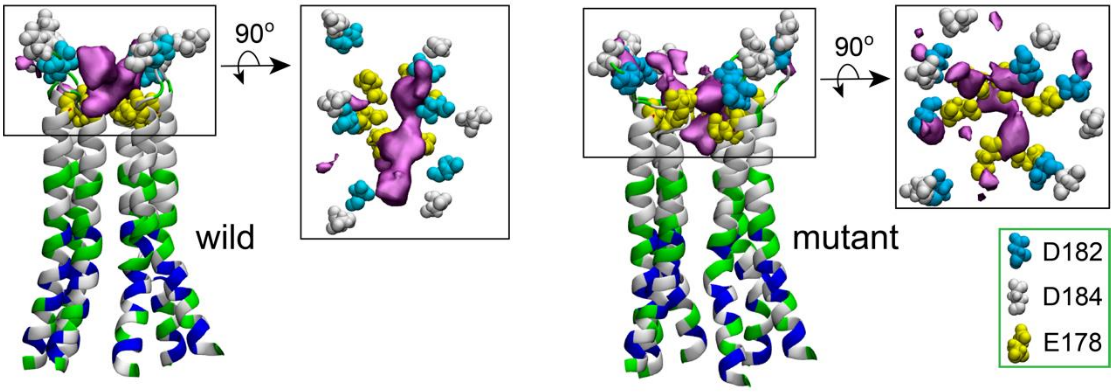

Figure S4. The Na^+ binding sites. The three binding sites E178, D182 and D184 are shown with VDW spheres. The isosurface of Na^+ ion densities are shown in pink with isosurface value of 0.02. Only residues from W148 to D184 are shown in NewCartoon and colored by residue types for clarity. Blue, basic residues; red, acidic residues; green, polar residues; white, nonpolar residues.

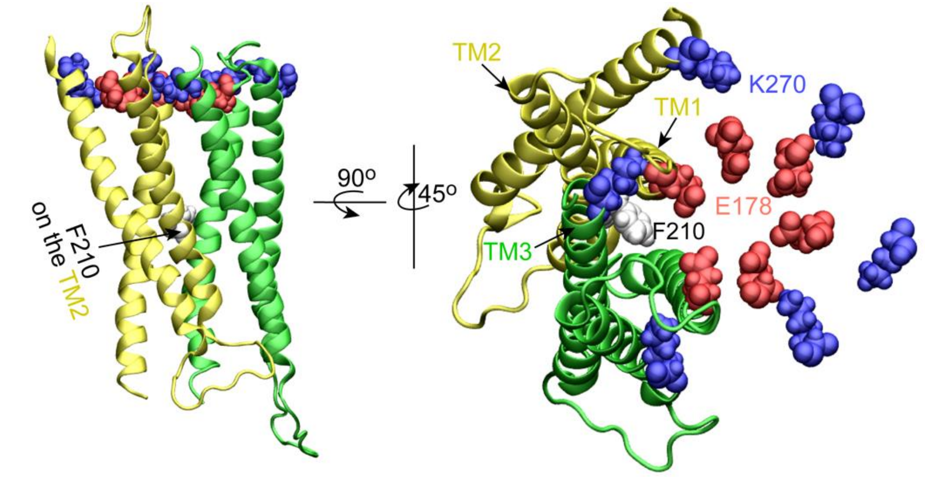

Figure S5. The location of F210 and the dilation of the SF. Two subunits are shown with yellow and green NewCartoon, respectively. Only the TM1, TM2 and TM3 are shown for clarity. F210 on the TM2 is shown with white VDW spheres. K270 on TM3 and E178 (the SF) on TM1 in all of the six subunits are shown with blue and red VDW spheres, respectively.
